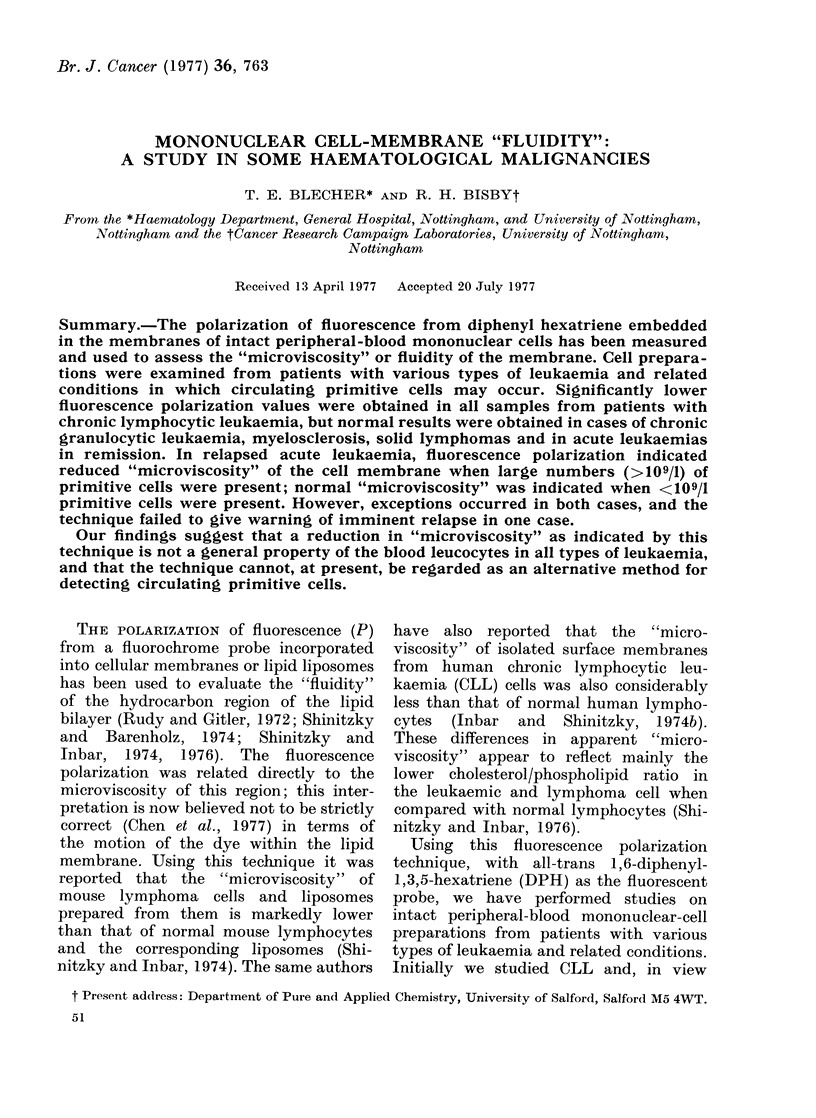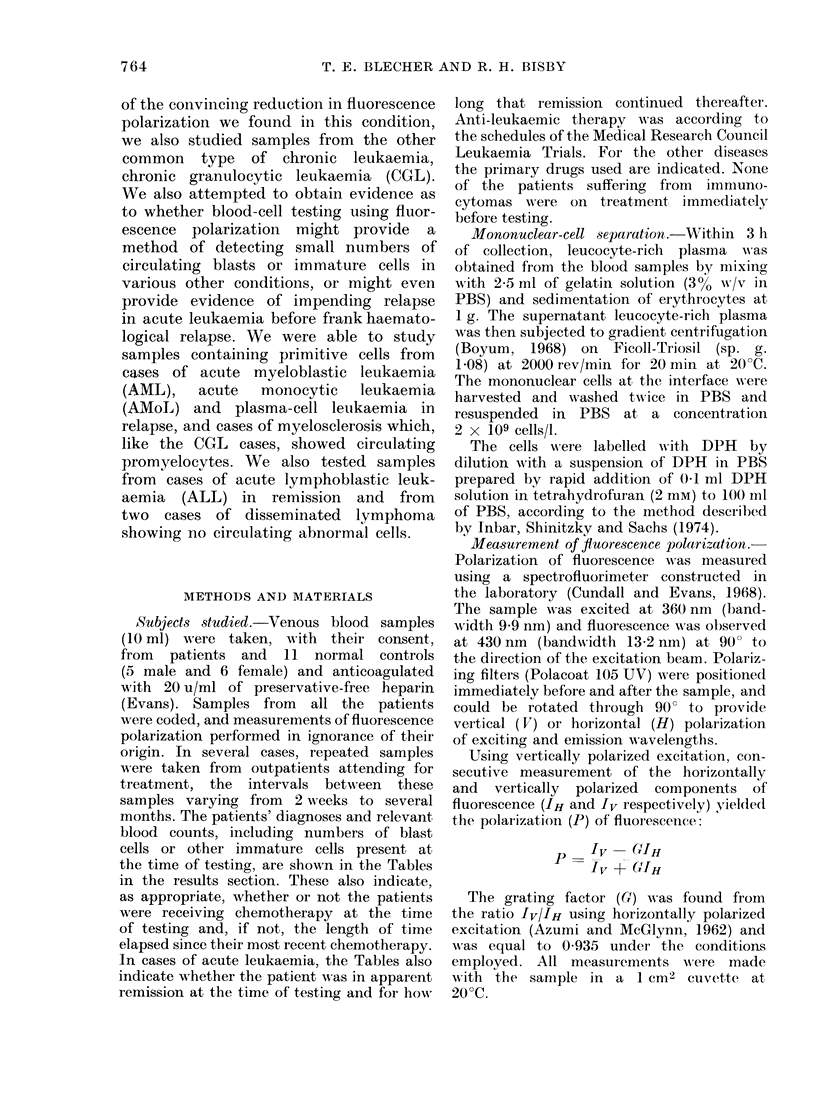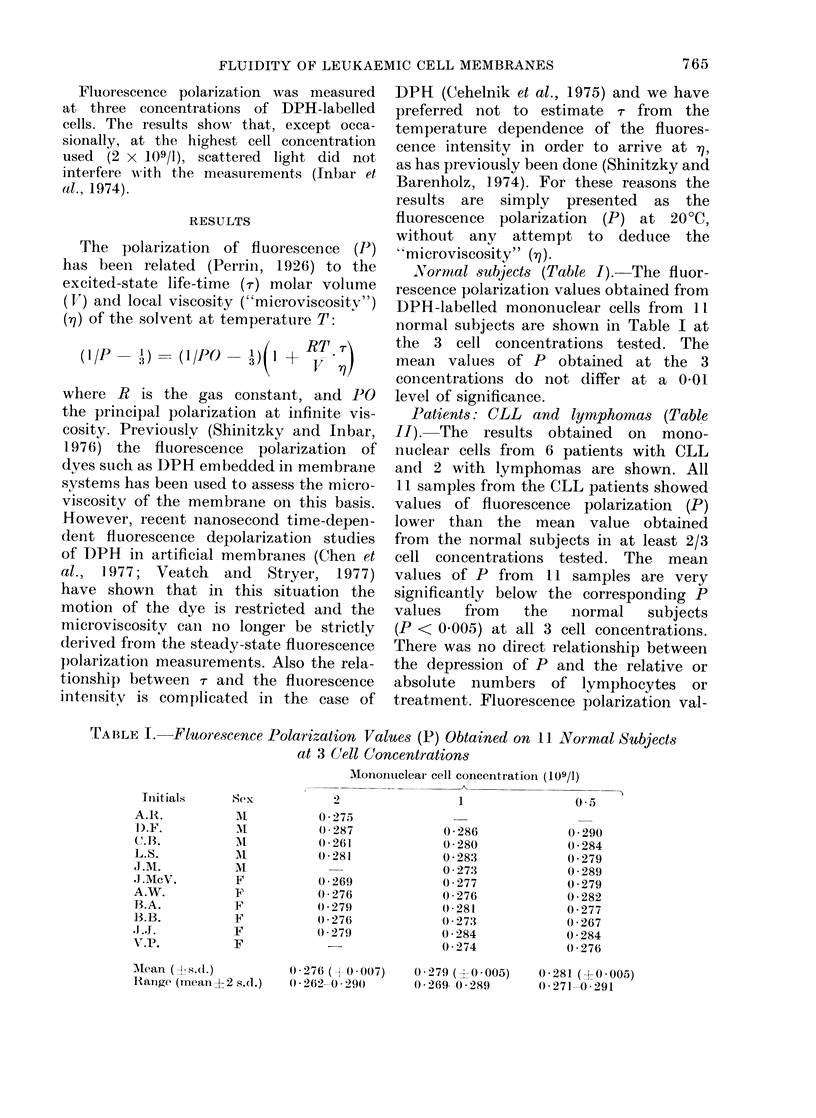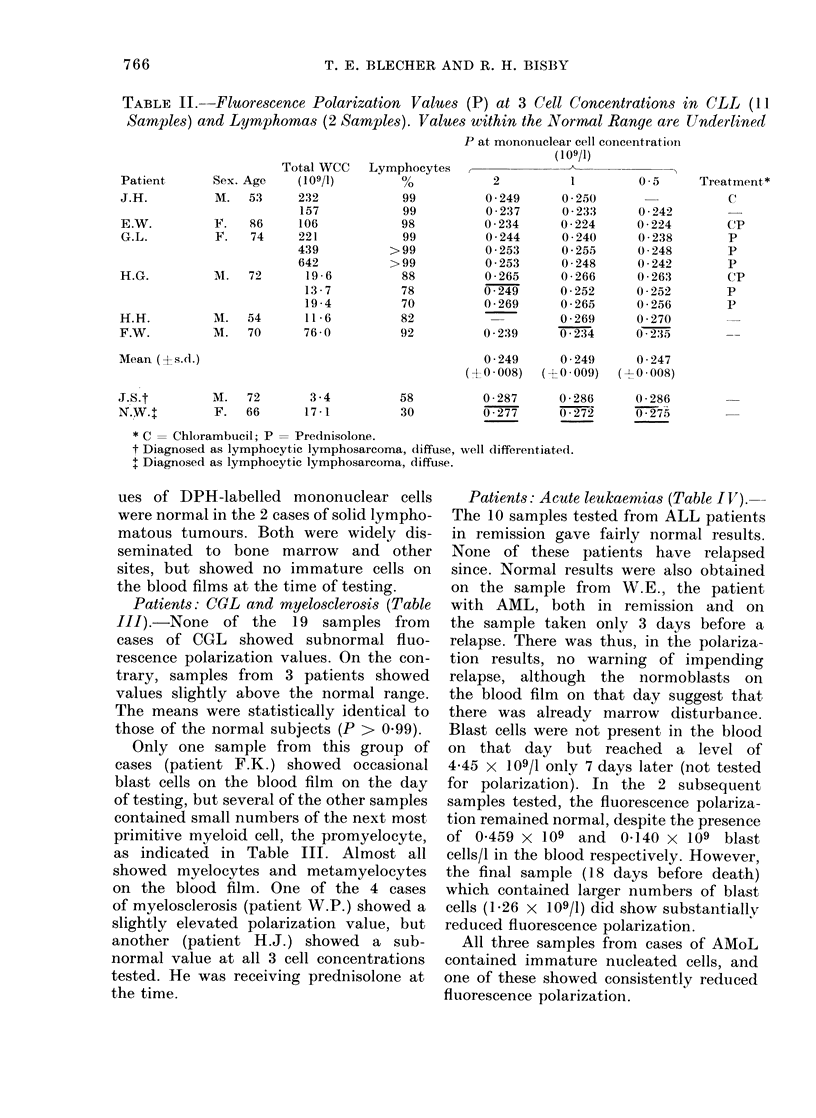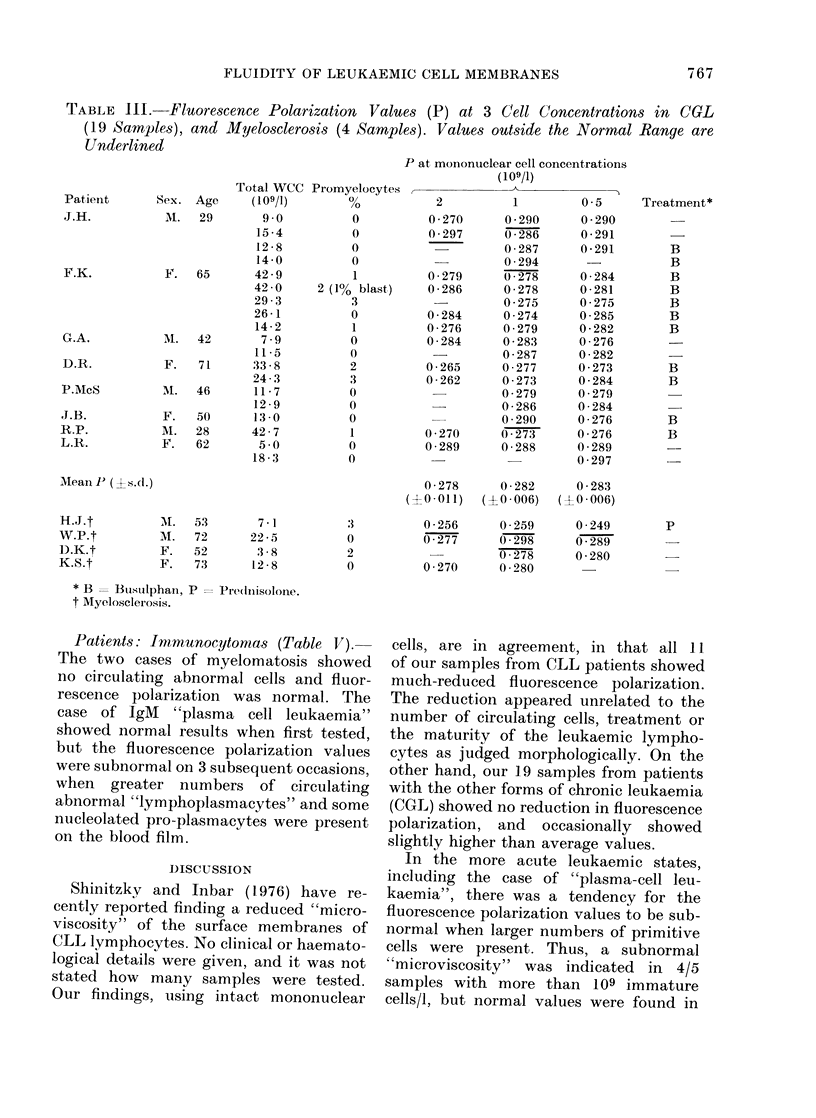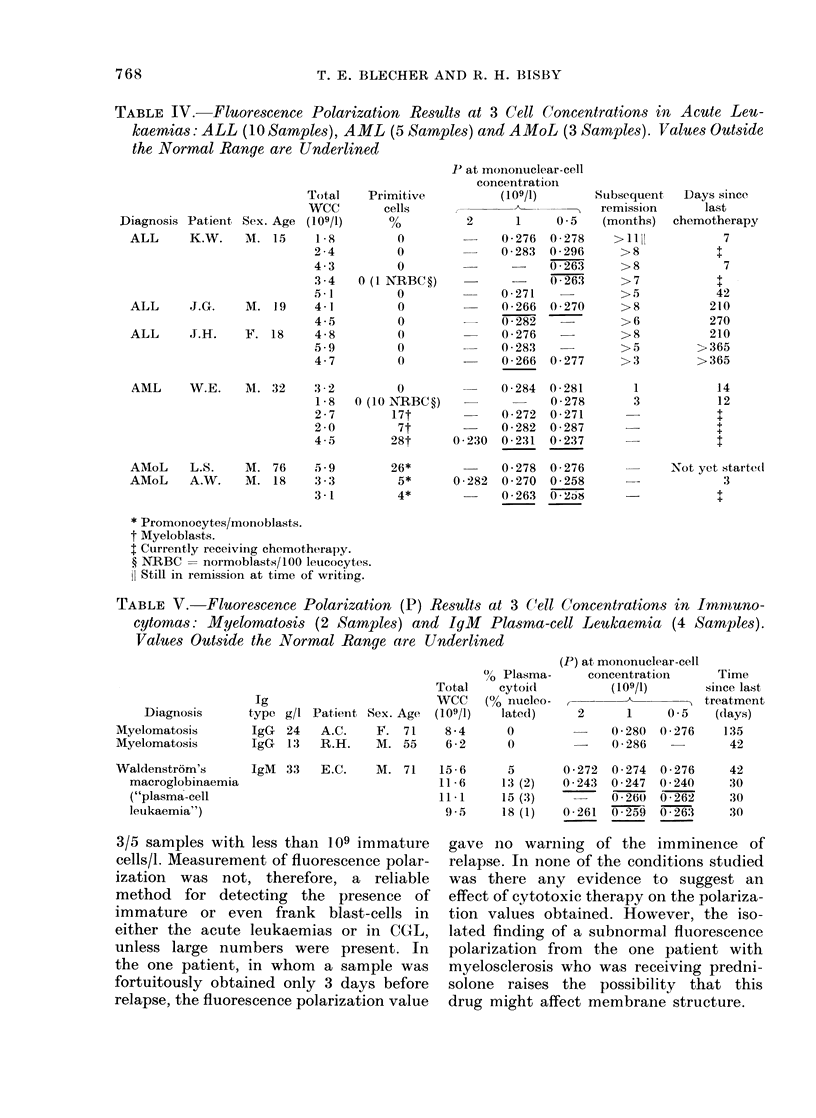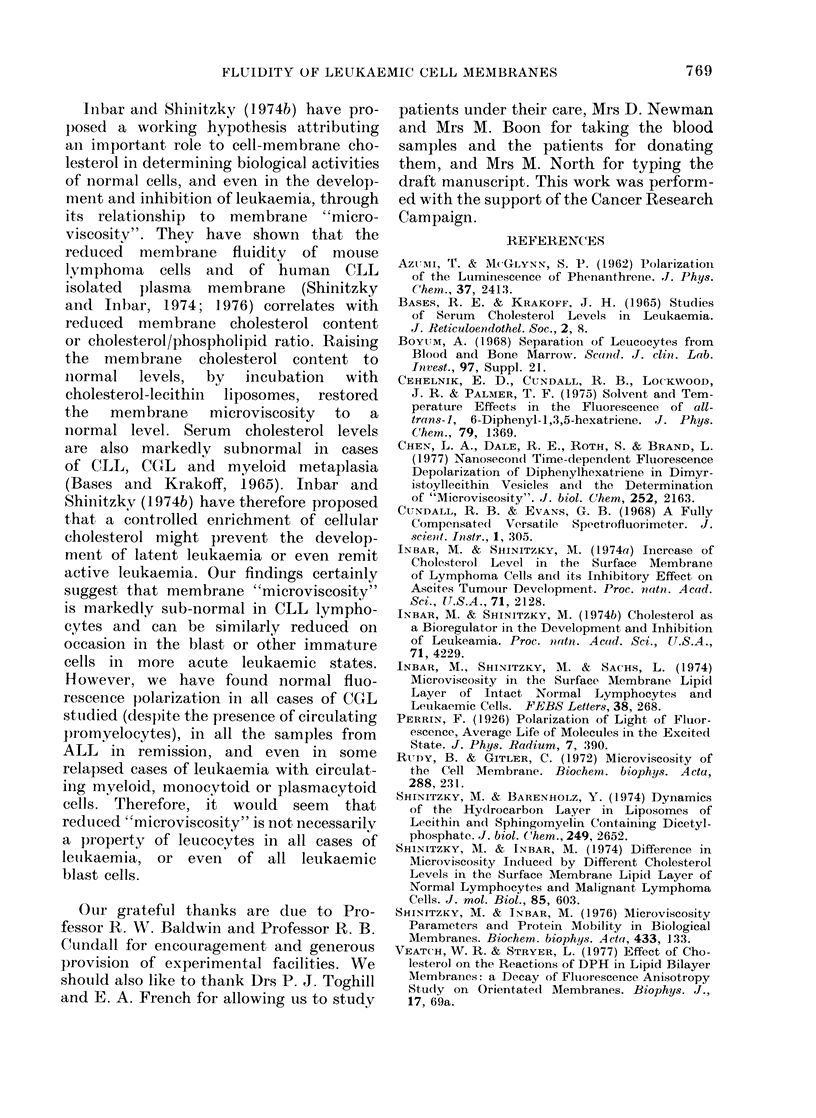# Mononuclear cell-membrane "fluidity": a study in some haematological malignancies.

**DOI:** 10.1038/bjc.1977.259

**Published:** 1977-12

**Authors:** T. E. Blecher, R. H. Bisby

## Abstract

The polarization of fluorescence from diphenyl hexatriene embedded in the membranes of intact peripheral-blood mononuclear cells has been measured and used to assess the "microviscosity" or fluidity of the membrane. Cell preparations were examined from patients with various types of leukaemia and related conditions in which circulating primitive cells may occur. Significantly lower fluorescence polarization values were obtained in all samples from patients with chronic lymphocytic leukaemia, but normal results were obtained in cases of chronic granulocytic leukaemia, myelosclerosis, solid lymphomas and in acute leukaemias in remission. In relapsed acute leukaemia, fluorescence polarization indicated reduced "microviscosity" of the cell membrane when large numbers (greater than 10(9)/1) of primitive cells were present; normal "microviscosity" was indicated when less than 10(9)/1 primitive cells were present. However, exceptions occurred in both cases, and the technique failed to give warning of imminent relapse in one case. Our findings suggest that a reduction in "microviscosity" as indicated by this technique is not a general property of the blood leucocytes in all types of leukaemia, and that the technique cannot, at present, be regarded as an alternative method for detecting circulating primitive cells.


*Br. J. Cancer* (1977) **36**, 763

### MONONUCLEAR CELL-MEMBRANE "FLUIDITY": A STUDY IN SOME HAEMATOLOGICAL MALIGNANCIES

T. E. BLECHER* AND R. H. BISBY†

*From the *Haematology Department, General Hospital, Nottingham, and University of Nottingham, Nottingham and the †Cancer Research Campaign Laboratories, University of Nottingham, Nottingham*

Received 13 April 1977 Accepted 20 July 1977

**Summary.—The polarization of fluorescence from diphenyl hexatriene embedded in the membranes of intact peripheral-blood mononuclear cells has been measured and used to assess the "microviscosity" or fluidity of the membrane. Cell preparations were examined from patients with various types of leukaemia and related conditions in which circulating primitive cells may occur. Significantly lower fluorescence polarization values were obtained in all samples from patients with chronic lymphocytic leukaemia, but normal results were obtained in cases of chronic granulocytic leukaemia, myelosclerosis, solid lymphomas and in acute leukaemias in remission. In relapsed acute leukaemia, fluorescence polarization indicated reduced "microviscosity" of the cell membrane when large numbers ($>10^9/l$) of primitive cells were present; normal "microviscosity" was indicated when $<10^9/l$ primitive cells were present. However, exceptions occurred in both cases, and the technique failed to give warning of imminent relapse in one case.**

**Our findings suggest that a reduction in "microviscosity" as indicated by this technique is not a general property of the blood leucocytes in all types of leukaemia, and that the technique cannot, at present, be regarded as an alternative method for detecting circulating primitive cells.**

THE POLARIZATION of fluorescence ($P$) from a fluorochrome probe incorporated into cellular membranes or lipid liposomes has been used to evaluate the "fluidity" of the hydrocarbon region of the lipid bilayer (Rudy and Gitler, 1972; Shinitzky and Barenholz, 1974; Shinitzky and Inbar, 1974, 1976). The fluorescence polarization was related directly to the microviscosity of this region; this interpretation is now believed not to be strictly correct (Chen *et al.*, 1977) in terms of the motion of the dye within the lipid membrane. Using this technique it was reported that the "microviscosity" of mouse lymphoma cells and liposomes prepared from them is markedly lower than that of normal mouse lymphocytes and the corresponding liposomes (Shinitzky and Inbar, 1974). The same authors have also reported that the "microviscosity" of isolated surface membranes from human chronic lymphocytic leukaemia (CLL) cells was also considerably less than that of normal human lymphocytes (Inbar and Shinitzky, 1974*b*). These differences in apparent "microviscosity" appear to reflect mainly the lower cholesterol/phospholipid ratio in the leukaemic and lymphoma cell when compared with normal lymphocytes (Shinitzky and Inbar, 1976).

Using this fluorescence polarization technique, with all-trans 1,6-diphenyl-1,3,5-hexatriene (DPH) as the fluorescent probe, we have performed studies on intact peripheral-blood mononuclear-cell preparations from patients with various types of leukaemia and related conditions. Initially we studied CLL and, in view

† Present address: Department of Pure and Applied Chemistry, University of Salford, Salford M5 4WT.

of the convincing reduction in fluorescence polarization we found in this condition, we also studied samples from the other common type of chronic leukaemia, chronic granulocytic leukaemia (CGL). We also attempted to obtain evidence as to whether blood-cell testing using fluorescence polarization might provide a method of detecting small numbers of circulating blasts or immature cells in various other conditions, or might even provide evidence of impending relapse in acute leukaemia before frank haematological relapse. We were able to study samples containing primitive cells from cases of acute myeloblastic leukaemia (AML), acute monocytic leukaemia (AMoL) and plasma-cell leukaemia in relapse, and cases of myelosclerosis which, like the CGL cases, showed circulating promyelocytes. We also tested samples from cases of acute lymphoblastic leukaemia (ALL) in remission and from two cases of disseminated lymphoma showing no circulating abnormal cells.

### METHODS AND MATERIALS

*Subjects studied.*—Venous blood samples (10 ml) were taken, with their consent, from patients and 11 normal controls (5 male and 6 female) and anticoagulated with 20 u/ml of preservative-free heparin (Evans). Samples from all the patients were coded, and measurements of fluorescence polarization performed in ignorance of their origin. In several cases, repeated samples were taken from outpatients attending for treatment, the intervals between these samples varying from 2 weeks to several months. The patients' diagnoses and relevant blood counts, including numbers of blast cells or other immature cells present at the time of testing, are shown in the Tables in the results section. These also indicate, as appropriate, whether or not the patients were receiving chemotherapy at the time of testing and, if not, the length of time elapsed since their most recent chemotherapy. In cases of acute leukaemia, the Tables also indicate whether the patient was in apparent remission at the time of testing and for how long that remission continued thereafter. Anti-leukaemic therapy was according to the schedules of the Medical Research Council Leukaemia Trials. For the other diseases the primary drugs used are indicated. None of the patients suffering from immunocytomas were on treatment immediately before testing.

*Mononuclear-cell separation.*—Within 3 h of collection, leucocyte-rich plasma was obtained from the blood samples by mixing with 2·5 ml of gelatin solution (3% w/v in PBS) and sedimentation of erythrocytes at 1 g. The supernatant leucocyte-rich plasma was then subjected to gradient centrifugation (Boyum, 1968) on Ficoll-Triosil (sp. g. 1·08) at 2000 rev/min for 20 min at 20°C. The mononuclear cells at the interface were harvested and washed twice in PBS and resuspended in PBS at a concentration $2 \times 10^9$ cells/l.

The cells were labelled with DPH by dilution with a suspension of DPH in PBS prepared by rapid addition of 0·1 ml DPH solution in tetrahydrofuran (2 mM) to 100 ml of PBS, according to the method described by Inbar, Shinitzky and Sachs (1974).

*Measurement of fluorescence polarization.*—Polarization of fluorescence was measured using a spectrofluorimeter constructed in the laboratory (Cundall and Evans, 1968). The sample was excited at 360 nm (bandwidth 9·9 nm) and fluorescence was observed at 430 nm (bandwidth 13·2 nm) at 90° to the direction of the excitation beam. Polarizing filters (Polacoat 105 UV) were positioned immediately before and after the sample, and could be rotated through 90° to provide vertical ($V$) or horizontal ($H$) polarization of exciting and emission wavelengths.

Using vertically polarized excitation, consecutive measurement of the horizontally and vertically polarized components of fluorescence ($I_H$ and $I_V$ respectively) yielded the polarization ($P$) of fluorescence:

$$P = \frac{I_V - GI_H}{I_V + GI_H}$$

The grating factor ($G$) was found from the ratio $I_V/I_H$ using horizontally polarized excitation (Azumi and McGlynn, 1962) and was equal to 0·935 under the conditions employed. All measurements were made with the sample in a 1 cm$^2$ cuvette at 20°C.

Fluorescence polarization was measured at three concentrations of DPH-labelled cells. The results show that, except occasionally, at the highest cell concentration used ($2 \times 10^9$/l), scattered light did not interfere with the measurements (Inbar *et al.*, 1974).

### RESULTS

The polarization of fluorescence ($P$) has been related (Perrin, 1926) to the excited-state life-time ($\tau$) molar volume ($V$) and local viscosity ("microviscosity") ($\eta$) of the solvent at temperature $T$:

$$(1/P - \tfrac{1}{3}) = (1/PO - \tfrac{1}{3})\left(1 + \frac{RT}{V} \cdot \frac{\tau}{\eta}\right)$$

where $R$ is the gas constant, and $PO$ the principal polarization at infinite viscosity. Previously (Shinitzky and Inbar, 1976) the fluorescence polarization of dyes such as DPH embedded in membrane systems has been used to assess the microviscosity of the membrane on this basis. However, recent nanosecond time-dependent fluorescence depolarization studies of DPH in artificial membranes (Chen *et al.*, 1977; Veatch and Stryer, 1977) have shown that in this situation the motion of the dye is restricted and the microviscosity can no longer be strictly derived from the steady-state fluorescence polarization measurements. Also the relationship between $\tau$ and the fluorescence intensity is complicated in the case of DPH (Cehelnik *et al.*, 1975) and we have preferred not to estimate $\tau$ from the temperature dependence of the fluorescence intensity in order to arrive at $\eta$, as has previously been done (Shinitzky and Barenholz, 1974). For these reasons the results are simply presented as the fluorescence polarization ($P$) at 20°C, without any attempt to deduce the "microviscosity" ($\eta$).

*Normal subjects* (*Table I*).—The fluorrescence polarization values obtained from DPH-labelled mononuclear cells from 11 normal subjects are shown in Table I at the 3 cell concentrations tested. The mean values of $P$ obtained at the 3 concentrations do not differ at a 0·01 level of significance.

*Patients: CLL and lymphomas* (*Table II*).—The results obtained on mononuclear cells from 6 patients with CLL and 2 with lymphomas are shown. All 11 samples from the CLL patients showed values of fluorescence polarization ($P$) lower than the mean value obtained from the normal subjects in at least 2/3 cell concentrations tested. The mean values of $P$ from 11 samples are very significantly below the corresponding $P$ values from the normal subjects ($P < 0·005$) at all 3 cell concentrations. There was no direct relationship between the depression of $P$ and the relative or absolute numbers of lymphocytes or treatment. Fluorescence polarization val-

TABLE I.—*Fluorescence Polarization Values* (P) *Obtained on* 11 *Normal Subjects at* 3 *Cell Concentrations*

| Initials | Sex | Mononuclear cell concentration ($10^9$/l) 2 | 1 | 0·5 |
|---|---|---|---|---|
| A.R. | M | 0·275 | — | — |
| D.F. | M | 0·287 | 0·286 | 0·290 |
| C.B. | M | 0·261 | 0·280 | 0·284 |
| L.S. | M | 0·281 | 0·283 | 0·279 |
| J.M. | M | — | 0·273 | 0·289 |
| J.McV. | F | 0·269 | 0·277 | 0·279 |
| A.W. | F | 0·276 | 0·276 | 0·282 |
| B.A. | F | 0·279 | 0·281 | 0·277 |
| B.B. | F | 0·276 | 0·273 | 0·267 |
| J.J. | F | 0·279 | 0·284 | 0·284 |
| V.P. | F | — | 0·274 | 0·276 |
| Mean (±s.d.) | | 0·276 (±0·007) | 0·279 (±0·005) | 0·281 (±0·005) |
| Range (mean±2 s.d.) | | 0·262–0·290 | 0·269–0·289 | 0·271–0·291 |

TABLE II.—*Fluorescence Polarization Values* (P) *at* 3 *Cell Concentrations in CLL* (11 *Samples*) *and Lymphomas* (2 *Samples*). *Values within the Normal Range are Underlined*

| Patient | Sex. | Age | Total WCC ($10^9$/l) | Lymphocytes % | *P* at mononuclear cell concentration ($10^9$/l) 2 | 1 | 0·5 | Treatment* |
|---|---|---|---|---|---|---|---|---|
| J.H. | M. | 53 | 232 | 99 | 0·249 | 0·250 | — | C |
| | | | 157 | 99 | 0·237 | 0·233 | 0·242 | — |
| E.W. | F. | 86 | 106 | 98 | 0·234 | 0·224 | 0·224 | CP |
| G.L. | F. | 74 | 221 | 99 | 0·244 | 0·240 | 0·238 | P |
| | | | 439 | >99 | 0·253 | 0·255 | 0·248 | P |
| | | | 642 | >99 | 0·253 | 0·248 | 0·242 | P |
| H.G. | M. | 72 | 19·6 | 88 | 0·265 | 0·266 | 0·263 | CP |
| | | | 13·7 | 78 | 0·249 | 0·252 | 0·252 | P |
| | | | 19·4 | 70 | 0·269 | 0·265 | 0·256 | P |
| H.H. | M. | 54 | 11·6 | 82 | — | 0·269 | 0·270 | — |
| F.W. | M. | 70 | 76·0 | 92 | 0·239 | 0·234 | 0·235 | — |
| Mean (±s.d.) | | | | | 0·249 (±0·008) | 0·249 (±0·009) | 0·247 (±0·008) | |
| J.S.† | M. | 72 | 3·4 | 58 | 0·287 | 0·286 | 0·286 | — |
| N.W.‡ | F. | 66 | 17·1 | 30 | 0·277 | 0·272 | 0·275 | — |

* C = Chlorambucil; P = Prednisolone.
† Diagnosed as lymphocytic lymphosarcoma, diffuse, well differentiated.
‡ Diagnosed as lymphocytic lymphosarcoma, diffuse.

ues of DPH-labelled mononuclear cells were normal in the 2 cases of solid lymphomatous tumours. Both were widely disseminated to bone marrow and other sites, but showed no immature cells on the blood films at the time of testing.

*Patients: CGL and myelosclerosis* (*Table III*).—None of the 19 samples from cases of CGL showed subnormal fluorescence polarization values. On the contrary, samples from 3 patients showed values slightly above the normal range. The means were statistically identical to those of the normal subjects ($P > 0{\cdot}99$).

Only one sample from this group of cases (patient F.K.) showed occasional blast cells on the blood film on the day of testing, but several of the other samples contained small numbers of the next most primitive myeloid cell, the promyelocyte, as indicated in Table III. Almost all showed myelocytes and metamyelocytes on the blood film. One of the 4 cases of myelosclerosis (patient W.P.) showed a slightly elevated polarization value, but another (patient H.J.) showed a subnormal value at all 3 cell concentrations tested. He was receiving prednisolone at the time.

*Patients: Acute leukaemias* (*Table IV*).—The 10 samples tested from ALL patients in remission gave fairly normal results. None of these patients have relapsed since. Normal results were also obtained on the sample from W.E., the patient with AML, both in remission and on the sample taken only 3 days before a relapse. There was thus, in the polarization results, no warning of impending relapse, although the normoblasts on the blood film on that day suggest that there was already marrow disturbance. Blast cells were not present in the blood on that day but reached a level of $4{\cdot}45 \times 10^9$/l only 7 days later (not tested for polarization). In the 2 subsequent samples tested, the fluorescence polarization remained normal, despite the presence of $0{\cdot}459 \times 10^9$ and $0{\cdot}140 \times 10^9$ blast cells/l in the blood respectively. However, the final sample (18 days before death) which contained larger numbers of blast cells ($1{\cdot}26 \times 10^9$/l) did show substantially reduced fluorescence polarization.

All three samples from cases of AMoL contained immature nucleated cells, and one of these showed consistently reduced fluorescence polarization.

TABLE III.—*Fluorescence Polarization Values* (P) *at* 3 *Cell Concentrations in CGL* (19 *Samples*), *and Myelosclerosis* (4 *Samples*). *Values outside the Normal Range are Underlined*

| Patient | Sex. | Age | Total WCC ($10^9$/l) | Promyelocytes % | *P* at mononuclear cell concentrations ($10^9$/l) 2 | 1 | 0·5 | Treatment* |
|---|---|---|---|---|---|---|---|---|
| J.H. | M. | 29 | 9·0 | 0 | 0·270 | 0·290 | 0·290 | — |
| | | | 15·4 | 0 | 0·297 | 0·286 | 0·291 | — |
| | | | 12·8 | 0 | — | 0·287 | 0·291 | B |
| | | | 14·0 | 0 | — | 0·294 | — | B |
| F.K. | F. | 65 | 42·9 | 1 | 0·279 | 0·278 | 0·284 | B |
| | | | 42·0 | 2 (1% blast) | 0·286 | 0·278 | 0·281 | B |
| | | | 29·3 | 3 | — | 0·275 | 0·275 | B |
| | | | 26·1 | 0 | 0·284 | 0·274 | 0·285 | B |
| | | | 14·2 | 1 | 0·276 | 0·279 | 0·282 | B |
| G.A. | M. | 42 | 7·9 | 0 | 0·284 | 0·283 | 0·276 | — |
| | | | 11·5 | 0 | — | 0·287 | 0·282 | — |
| D.R. | F. | 71 | 33·8 | 2 | 0·265 | 0·277 | 0·273 | B |
| | | | 24·3 | 3 | 0·262 | 0·273 | 0·284 | B |
| P.McS | M. | 46 | 11·7 | 0 | — | 0·279 | 0·279 | — |
| | | | 12·9 | 0 | — | 0·286 | 0·284 | — |
| J.B. | F. | 50 | 13·0 | 0 | — | 0·290 | 0·276 | B |
| R.P. | M. | 28 | 42·7 | 1 | 0·270 | 0·273 | 0·276 | B |
| L.R. | F. | 62 | 5·0 | 0 | 0·289 | 0·288 | 0·289 | — |
| | | | 18·3 | 0 | — | — | 0·297 | — |
| Mean *P* (±s.d.) | | | | | 0·278 (±0·011) | 0·282 (±0·006) | 0·283 (±0·006) | |
| H.J.† | M. | 53 | 7·1 | 3 | 0·256 | 0·259 | 0·249 | P |
| W.P.† | M. | 72 | 22·5 | 0 | 0·277 | 0·298 | 0·289 | — |
| D.K.† | F. | 52 | 3·8 | 2 | — | 0·278 | 0·280 | — |
| K.S.† | F. | 73 | 12·8 | 0 | 0·270 | 0·280 | — | — |

* B = Busulphan, P = Prednisolone.

† Myelosclerosis.

*Patients: Immunocytomas* (*Table V*).—The two cases of myelomatosis showed no circulating abnormal cells and fluorrescence polarization was normal. The case of IgM "plasma cell leukaemia" showed normal results when first tested, but the fluorescence polarization values were subnormal on 3 subsequent occasions, when greater numbers of circulating abnormal "lymphoplasmacytes" and some nucleolated pro-plasmacytes were present on the blood film.

### DISCUSSION

Shinitzky and Inbar (1976) have recently reported finding a reduced "microviscosity" of the surface membranes of CLL lymphocytes. No clinical or haematological details were given, and it was not stated how many samples were tested. Our findings, using intact mononuclear cells, are in agreement, in that all 11 of our samples from CLL patients showed much-reduced fluorescence polarization. The reduction appeared unrelated to the number of circulating cells, treatment or the maturity of the leukaemic lymphocytes as judged morphologically. On the other hand, our 19 samples from patients with the other forms of chronic leukaemia (CGL) showed no reduction in fluorescence polarization, and occasionally showed slightly higher than average values.

In the more acute leukaemic states, including the case of "plasma-cell leukaemia", there was a tendency for the fluorescence polarization values to be subnormal when larger numbers of primitive cells were present. Thus, a subnormal "microviscosity" was indicated in 4/5 samples with more than $10^9$ immature cells/l, but normal values were found in

TABLE IV.—*Fluorescence Polarization Results at 3 Cell Concentrations in Acute Leukaemias: ALL (10 Samples), AML (5 Samples) and AMoL (3 Samples). Values Outside the Normal Range are Underlined*

| Diagnosis | Patient | Sex. | Age | Total WCC (10⁹/l) | Primitive cells % | P at mononuclear-cell concentration (10⁹/l) 2 | 1 | 0·5 | Subsequent remission (months) | Days since last chemotherapy |
|---|---|---|---|---|---|---|---|---|---|---|
| ALL | K.W. | M. | 15 | 1·8 | 0 | — | 0·276 | 0·278 | >11‖ | 7 |
| | | | | 2·4 | 0 | — | 0·283 | 0·296 | >8 | ‡ |
| | | | | 4·3 | 0 | — | — | 0·263 | >8 | 7 |
| | | | | 3·4 | 0 (1 NRBC§) | — | — | 0·263 | >7 | ‡ |
| | | | | 5·1 | 0 | — | 0·271 | — | >5 | 42 |
| ALL | J.G. | M. | 19 | 4·1 | 0 | — | 0·266 | 0·270 | >8 | 210 |
| | | | | 4·5 | 0 | — | 0·282 | — | >6 | 270 |
| ALL | J.H. | F. | 18 | 4·8 | 0 | — | 0·276 | — | >8 | 210 |
| | | | | 5·9 | 0 | — | 0·283 | — | >5 | >365 |
| | | | | 4·7 | 0 | — | 0·266 | 0·277 | >3 | >365 |
| AML | W.E. | M. | 32 | 3·2 | 0 | — | 0·284 | 0·281 | 1 | 14 |
| | | | | 1·8 | 0 (10 NRBC§) | — | — | 0·278 | 3 | 12 |
| | | | | 2·7 | 17† | — | 0·272 | 0·271 | — | ‡ |
| | | | | 2·0 | 7† | — | 0·282 | 0·287 | — | ‡ |
| | | | | 4·5 | 28† | 0·230 | 0·231 | 0·237 | — | ‡ |
| AMoL | L.S. | M. | 76 | 5·9 | 26* | — | 0·278 | 0·276 | — | Not yet started |
| AMoL | A.W. | M. | 18 | 3·3 | 5* | 0·282 | 0·270 | 0·258 | — | 3 |
| | | | | 3·1 | 4* | — | 0·263 | 0·258 | — | ‡ |

* Promonocytes/monoblasts.
† Myeloblasts.
‡ Currently receiving chemotherapy.
§ NRBC = normoblasts/100 leucocytes.
‖ Still in remission at time of writing.

TABLE V.—*Fluorescence Polarization (P) Results at 3 Cell Concentrations in Immunocytomas: Myelomatosis (2 Samples) and IgM Plasma-cell Leukaemia (4 Samples). Values Outside the Normal Range are Underlined*

| Diagnosis | Ig type | g/l | Patient | Sex. | Age | Total WCC (10⁹/l) | % Plasma-cytoid (% nucleolated) | (P) at mononuclear-cell concentration (10⁹/l) 2 | 1 | 0·5 | Time since last treatment (days) |
|---|---|---|---|---|---|---|---|---|---|---|---|
| Myelomatosis | IgG | 24 | A.C. | F. | 71 | 8·4 | 0 | — | 0·280 | 0·276 | 135 |
| Myelomatosis | IgG | 13 | R.H. | M. | 55 | 6·2 | 0 | — | 0·286 | — | 42 |
| Waldenström's | IgM | 33 | E.C. | M. | 71 | 15·6 | 5 | 0·272 | 0·274 | 0·276 | 42 |
| macroglobinaemia | | | | | | 11·6 | 13 (2) | 0·243 | 0·247 | 0·240 | 30 |
| ("plasma-cell | | | | | | 11·1 | 15 (3) | — | 0·260 | 0·262 | 30 |
| leukaemia") | | | | | | 9·5 | 18 (1) | 0·261 | 0·259 | 0·263 | 30 |

3/5 samples with less than $10^9$ immature cells/l. Measurement of fluorescence polarization was not, therefore, a reliable method for detecting the presence of immature or even frank blast-cells in either the acute leukaemias or in CGL, unless large numbers were present. In the one patient, in whom a sample was fortuitously obtained only 3 days before relapse, the fluorescence polarization value gave no warning of the imminence of relapse. In none of the conditions studied was there any evidence to suggest an effect of cytotoxic therapy on the polarization values obtained. However, the isolated finding of a subnormal fluorescence polarization from the one patient with myelosclerosis who was receiving prednisolone raises the possibility that this drug might affect membrane structure.

Inbar and Shinitzky (1974*b*) have proposed a working hypothesis attributing an important role to cell-membrane cholesterol in determining biological activities of normal cells, and even in the development and inhibition of leukaemia, through its relationship to membrane "microviscosity". They have shown that the reduced membrane fluidity of mouse lymphoma cells and of human CLL isolated plasma membrane (Shinitzky and Inbar, 1974; 1976) correlates with reduced membrane cholesterol content or cholesterol/phospholipid ratio. Raising the membrane cholesterol content to normal levels, by incubation with cholesterol-lecithin liposomes, restored the membrane microviscosity to a normal level. Serum cholesterol levels are also markedly subnormal in cases of CLL, CGL and myeloid metaplasia (Bases and Krakoff, 1965). Inbar and Shinitzky (1974*b*) have therefore proposed that a controlled enrichment of cellular cholesterol might prevent the development of latent leukaemia or even remit active leukaemia. Our findings certainly suggest that membrane "microviscosity" is markedly sub-normal in CLL lymphocytes and can be similarly reduced on occasion in the blast or other immature cells in more acute leukaemic states. However, we have found normal fluorescence polarization in all cases of CGL studied (despite the presence of circulating promyelocytes), in all the samples from ALL in remission, and even in some relapsed cases of leukaemia with circulating myeloid, monocytoid or plasmacytoid cells. Therefore, it would seem that reduced "microviscosity" is not necessarily a property of leucocytes in all cases of leukaemia, or even of all leukaemic blast cells.

Our grateful thanks are due to Professor R. W. Baldwin and Professor R. B. Cundall for encouragement and generous provision of experimental facilities. We should also like to thank Drs P. J. Toghill and E. A. French for allowing us to study patients under their care, Mrs D. Newman and Mrs M. Boon for taking the blood samples and the patients for donating them, and Mrs M. North for typing the draft manuscript. This work was performed with the support of the Cancer Research Campaign.

### REFERENCES

AZUMI, T. & McGLYNN, S. P. (1962) Polarization of the Luminescence of Phenanthrene. *J. Phys. Chem.*, **37**, 2413.

BASES, R. E. & KRAKOFF, J. H. (1965) Studies of Serum Cholesterol Levels in Leukaemia. *J. Reticuloendothel. Soc.*, **2**, 8.

BOYUM, A. (1968) Separation of Leucocytes from Blood and Bone Marrow. *Scand. J. clin. Lab. Invest.*, **97**, Suppl. 21.

CEHELNIK, E. D., CUNDALL, R. B., LOCKWOOD, J. R. & PALMER, T. F. (1975) Solvent and Temperature Effects in the Fluorescence of *all-trans-1*, 6-Diphenyl-1,3,5-hexatriene. *J. Phys. Chem.*, **79**, 1369.

CHEN, L. A., DALE, R. E., ROTH, S. & BRAND, L. (1977) Nanosecond Time-dependent Fluorescence Depolarization of Diphenylhexatriene in Dimyristoyllecithin Vesicles and the Determination of "Microviscosity". *J. biol. Chem*, **252**, 2163.

CUNDALL, R. B. & EVANS, G. B. (1968) A Fully Compensated Versatile Spectrofluorimeter. *J. scient. Instr.*, **1**, 305.

INBAR, M. & SHINITZKY, M. (1974*a*) Increase of Cholesterol Level in the Surface Membrane of Lymphoma Cells and its Inhibitory Effect on Ascites Tumour Development. *Proc. natn. Acad. Sci., U.S.A.*, **71**, 2128.

INBAR, M. & SHINITZKY, M. (1974*b*) Cholesterol as a Bioregulator in the Development and Inhibition of Leukeamia. *Proc. natn. Acad. Sci., U.S.A.*, **71**, 4229.

INBAR, M., SHINITZKY, M. & SACHS, L. (1974) Microviscosity in the Surface Membrane Lipid Layer of Intact Normal Lymphocytes and Leukaemic Cells. *FEBS Letters*, **38**, 268.

PERRIN, F. (1926) Polarization of Light of Fluorescence, Average Life of Molecules in the Excited State. *J. Phys. Radium*, **7**, 390.

RUDY, B. & GITLER, C. (1972) Microviscosity of the Cell Membrane. *Biochem. biophys. Acta*, **288**, 231.

SHINITZKY, M. & BARENHOLZ, Y. (1974) Dynamics of the Hydrocarbon Layer in Liposomes of Lecithin and Sphingomyelin Containing Dicetylphosphate. *J. biol. Chem.*, **249**, 2652.

SHINITZKY, M. & INBAR, M. (1974) Difference in Microviscosity Induced by Different Cholesterol Levels in the Surface Membrane Lipid Layer of Normal Lymphocytes and Malignant Lymphoma Cells. *J. mol. Biol.*, **85**, 603.

SHINITZKY, M. & INBAR, M. (1976) Microviscosity Parameters and Protein Mobility in Biological Membranes. *Biochem. biophys. Acta*, **433**, 133.

VEATCH, W. R. & STRYER, L. (1977) Effect of Cholesterol on the Reactions of DPH in Lipid Bilayer Membranes: a Decay of Fluorescence Anisotropy Study on Orientated Membranes. *Biophys. J.*, **17**, 69a.